# Comparative Analysis of Anion Exchange Membrane Adsorbers for Endotoxin Removal During Ultrafiltration and Diafiltration Buffer Preparation

**DOI:** 10.1002/elsc.70029

**Published:** 2025-07-03

**Authors:** Sven Heimann, Andrew Vail, Sophie Muczenski, Alexander Faude

**Affiliations:** ^1^ Rentschler Biopharma SE Laupheim Germany; ^2^ Solventum Corporation Purification and Filtration Business St. Paul Minnesota USA

**Keywords:** chromatography, endotoxin, guanidinium, membrane, single‐use

## Abstract

During the manufacturing of drug substances (DS), endotoxins are a commonly monitored contaminant and checked to ensure the safety and quality of the final product. In this study, we investigated endotoxin removal by commercial anion exchange membrane adsorbers during buffer preparation for an ultrafiltration and diafiltration (UFDF) unit operation as a risk mitigation strategy to meet DS endotoxin specifications. A bracketing design study was used to compare two quaternary amine (Q) modified membranes and one guanidinium functionalized hybrid adsorber for endotoxin removal from spiked buffers representing UFDF start and diafiltration buffer matrices. For the UFDF start buffer, Q‐based membrane adsorbers were effective at the removal of endotoxin to the limit of detection in low ionic strength conditions, with one adsorber effective up to 24 mS/cm. Switching to a more complex UFDF diafiltration buffer, Q‐based membrane adsorbers were impacted by additional buffer components. The guanidinium‐based hybrid membrane adsorber demonstrated endotoxin reduction to the limit of detection from both buffer matrices, showing removal across a wide pH range (4.7–8.3) and conductivity as high as 43 mS/cm. These results demonstrate an operational window for buffers and selected membrane adsorbers to mitigate risk by limiting endotoxin contamination prior to UFDF operations in pharmaceutical applications.

**Abbreviations**
AEX: anion exchange
API: active pharmaceutical ingredient
cm: centimeter
DEAE: diethylamino ethyl
DoE: design of experiments
DP: drug product
DS: drug substance
EU: endotoxin units
G: guanidinium
G1: 3M(TM) Polisher ST
L: liter
L/m^2^
: liters per meter squared
LMH: liters per meter squared per hour
mL: milliliter
mM: millimolar
mS/cm: milli Siemens per centimeter
MV: membrane volume
NaCl: sodium chloride
NaOH: sodium hydroxide
PA: primary amine
PBB: polymyxin B
PLL: poly‐L‐lysine
Q: quaternary ammonium
Q1: Cytiva Mustang(TM) E
Q2: Sartorius Sartobind(R) Q
UFDF: ultrafiltration and diafiltration

## 1 Introduction

Endotoxin control is an essential part during the production and manufacturing of an active pharmaceutical ingredient or API [1, 2]. Endotoxins are components of the outer cell membranes of nearly all Gram‐negative bacteria and are known to cause immunological responses after exposure to solutions containing endotoxin [3, 4, 5]. Sources of endotoxins during manufacturing of an API include raw input materials (e.g., water, chemicals, etc.), production equipment (e.g., reactors, filters, resins, etc.), and other processing components (e.g., tubing, containers, connectors, etc.). These impurities can contaminate process intermediates during different unit operations to manufacture drug substances (DS) and drug products (DP), like buffers used for elution from chromatographic processes, suspension of therapeutic protein‐based drugs, and diafiltration. Endotoxin introduction from buffer components associated with the ultrafiltration/diafiltration (UFDF) unit operation is especially concerning, as this is typically the last step of DS production, increasing the risk of patient safety.

**Summary**

- Endotoxin is an undesirable impurity during drug manufacturing, and mitigation strategies during late‐stage production of drug substances can be applied to reduce contamination risk. Potential concentration of endotoxin from premade buffers could occur during ultrafiltration and diafiltration (UFDF) operation.
- This comparative study investigated commercially available anion exchange (AEX) membrane adsorbers for the capability to remove low concentrations of endotoxin from UFDF start and diafiltration buffer matrices as a risk mitigation strategy. Bracketing design studies showed differences between the quaternary ammonium and guanidinium membrane adsorbers.
- For a practical application, the guanidinium‐based membrane adsorber was processed up to 10,000 L/m^2^ at a flux of 3,600 LMH with a UFDF start buffer and the resulting filtrate contained endotoxin below the limit of detections (<0.1 EU/mL).

The endotoxin release specification of DS as a final product in API production is based on the target dosage of the API and authority guidelines, such as United States Pharmacopeia harmonized standard <85> or the European Pharmacopoeia harmonized standard 5.0 (Version 2.6.14) bacterial endotoxins test, in dependency of the route of administration. The resulting DS endotoxin specification may be evaluated as risk for batch release, taking potential concentration of endotoxins in UFDF steps into account, considering worst‐case calculations based on buffer raw material specifications. A risk mitigation strategy to meet the DS endotoxin specification for this stage is based on three potential approaches of endotoxin reduction: (i) during buffer preparation; (ii) during feed of the diafiltration buffer in the retentate vessel and final UFDF step; and (iii) product intermediates.

Common endotoxin removal methods include techniques such as size exclusion by ultrafiltration, chromatographic adsorption (e.g. anion exchange, affinity, hydrophobic, and multimodal), or phase separation [1, 6, 7, 8, 9, 10, 11]. Solvent extraction or phase separation benefits small‐scale sample preparation, but organic solvent handling and processing of large volumes is not ideal when simpler options are available. Ultrafiltration could be utilized, but requires additional equipment and processing time. Adsorption techniques are frequently employed to remove endotoxin, as anion exchange and multimodal anion exchange ligand chemistry can bind to endotoxin through the negatively charged moieties of endotoxins [12, 13, 14, 15]. Suppliers of resins offer numerous chemical ligands, but this format requires packing of chromatography columns, qualification of resin packing, cleaning validation, and diffusive properties inherently increasing cost and processing time. Membrane adsorbers offer a distinct advantage over resins as they offer single‐use options with faster processing times, reduced investment, and decreased buffer consumption, but lack available commercial options in terms of chemical ligands reported to remove endotoxin.

Membrane adsorbers functionalized with alpha‐amylase, deoxycholate, diethylaminoethyl (DEAE), histamine, histidine, poly(ethylenimine), poly‐L‐lysine (PLL), polymyxin B (PMB), primary amine (PA), and quaternary ammonium (Q) have been shown to reduce endotoxin from protein, protein‐free, and phage‐containing solutions [16, 17, 18, 19, 20]. Studies with DEAE, histamine, histidine, PLL, and PMB ligands immobilized on sorbents exhibited high endotoxin removal from protein‐containing solutions but are only effective in low ionic strength conditions (<150 mM NaCl) [21]. Q ligand functionalized on a hydrophilic polyvinylidene‐based membrane showed high levels of endotoxin removal across a wide pH range, but was sensitive to NaCl concentrations above 150 mM [20]. A Q‐based functionalized nonwoven, 3M Emphaze AEX Hybrid Purifier, demonstrated endotoxin removal from phosphate‐buffered saline (137 mM NaCl and 2.7 mM KCl) containing proteins (https://www.solventum.com/content/dam/public/language‐masters/en/pfb/document/2020/Emphaze‐AEX‐Hybrid‐Purifier‐App‐Note‐02‐LR.pdf.coredownload.inline.pdf, accessed March 3, 2025) and bacterial lysate preparations [22]. Membrane modified with PA chemistry has demonstrated an increased binding capacity toward bovine serum albumin and phiX174 phage under higher conductivity environments compared to Q‐based membranes. This is due to the inherent properties of chemistry and charge density. Additionally, these membranes have shown endotoxin removal effectiveness up to 16.8 mS/cm (150 mM NaCl buffer solution) [19]. Although Q functional media might be impacted at higher salt concentrations, they may still be effective at removing residual endotoxin concentrations.

For this study, we compared three different flow‐through AEX membrane or hybrid adsorbers based on availability and suitability for API production, product size range, and previous manufacturer literature related to removal of endotoxin and other negatively charged impurities: 3M(TM) Polisher ST, Cytiva Mustang(TM) E and Sartorius Sartobind(R) Q. Product literature for Cytiva Mustang E (Q1) states it was designed for endotoxin removal from complex aqueous solutions, including high salt solutions, and has shown dynamic binding capacities of *Escherichia coli* endotoxin in saline up to 4 × 10^6^ EU/mL of media. Q1 membranes are manufactured with a proprietary polyethersulfone membrane modified with a highly cross‐linked quaternized amine charge polymer coating based on polyethyleneimine. Similarly, an application note reported Sartorius Sartobind Q (Q2) exemplified a 5‐log reduction of endotoxins from an enzyme‐containing buffer with a conductivity of range of 16.6–17.4 mS/cm (https://www.sartorius.com/download/1071402/sartobind‐endotoxin‐removal‐application‐note‐en‐b‐sl‐4040‐sa‐1‐data.pdf, accessed on March 3, 2025). A manufacturer validation guide indicates Q2 membranes are made from crosslinked, regenerated cellulose reinforced with non‐woven polyester and functional groups are linked to the entire surface of the macro‐porous membrane. 3M Polisher ST (G1) comprises a quaternary ammonium functional nonwoven, similar to 3M(TM) Emphaze AEX Hybrid Purifier, which has been demonstrated to reduce endotoxins in protein‐containing phosphate‐buffered saline solutions reported in a supplier application note, in addition to a guanidinium (G) modified nylon membrane [23]. Material characteristics of the tested filters are summarized in Table 1.

**TABLE 1:** Manufacturer specifications for chromatographic products.

Specifications	Cytiva Mustang E (Q1)	Sartorius Sartobind Q (Q2)	3M Polisher ST (G1)
Device	Acrodisc (25 mm)	Sartobind Q Pico	BC1
Filter media	Highly cross‐linked Q‐ligand surface modified polyethersulfone membrane	Q‐ligand on stabilized reinforced cellulose quaternary ammonium	Q polymer grafted to a polypropylene nonwoven; G‐functional polyamide membrane
Nominal pore size	0.2 µm	>3 µm	NA nonwoven; 0.8 µm membrane
Membrane bed volume (MV)	0.12 mL	0.08 mL	0.35 mL Q‐nonwoven 0.14 mL G‐membrane
Frontal surface area	NA	0.19 cm^2^	1 cm^2^
Absorptive area	NA	2.9 cm^2^	NA
Maximum operating pressure	5.5 bar at 21°C–24°C	6 bar at 20°C	3.4 bar (inlet) 2.4 bar (ΔP) 40°C
Recommended flow rates	1–4 mL/min (10–35 MV/min)	0.8–2.4 mL/min (10–30 MV/min)	1 mL/min (600 L/(m^2^*h))
Recommended preconditioning	1. >10 MV of 1 M NaOH 2. >10 MV of 1 M NaCl 3. >30 MV of equilibration buffer	1. 30 MV of 1 M NaOH 2. 50 MV of 1 M NaCl 3. 50 MV of equilibration buffer	5 mL (54 L/m^2^) of aqueous solution with conductivity ≥ 3 mS/cm
Endotoxin binding capacity	4 × 10^6^ EU/mL membrane in saline	1.25 × 10^7^ EU/mL membrane in PBS	NA
BSA dynamic binding capacity at 10% breakthrough	NA	0.8 mg/cm^2^ or 29 mg/mL from 20 mM Tris‐HCl, pH 7.5	BC1: 20 mg/cm^2^ from 25 mM Tris‐HCl, 50 mM NaCl, pH 8 G‐membrane: 7.7–12 mg/cm^2^ from 25 mM Tris‐HCl, 50 mM NaCl, pH 8

Abbreviations: G, guanidinium; MV, membrane volume, NA, not available; Q, quaternary ammonium.

These anion exchange chromatography solutions were studied in a buffer bracketing design utilizing a design of experiment (DoE) based approach and covered a broad range of nonspecific matrices used for UFDF starting buffers and diafiltration buffers. A UFDF step is often used at late‐stage production to adjust the API concentration and perform the matrix exchange against a defined excipient composition. The UFDF start and diafiltration buffer matrix should feature a low endotoxin level, as endotoxins may be concentrated following a UFDF step. The UFDF start buffer model is based on a generic buffer system for pH‐driven process steps. The pH and conductivity ranges covered different combinations of process steps, which precede the final UFDF step. In this investigation, components of a representative UFDF diafiltration buffer are aligned with opinions of formulation development experts at Rentschler Biopharma, while pH and conductivity ranges are chosen to include common formulation conditions. An ideal solution for a flow‐through anion exchange unit operation would have reliable endotoxin reduction over a broad range of pH and conductivity, available product size range to account for buffer volumes at development and production scale, reduced pre‐processing or conditioning steps, and integrated bioburden filtration function to avoid multiple filter processes.

## 2 Materials and Methods

This study was performed in several stages. In the first stage, the relevance of preconditioning flush steps was evaluated with the aim to reduce handling time at production scale. Following the first stage, the chromatographic solutions were examined for their endotoxin reduction within a design space comprising a representative UFDF start buffer and UFDF diafiltration buffer. Finally, the lead chromatographic anion exchanger was challenged by running at the maximum flux allowed, based on product specifications, with a safety factor of >10% to optimize this application at production scale.

### 2.1 Single Use Chromatography

The anion exchange chromatographic products used in this study were Cytiva Mustang E Acrodisc, Sartorius Sartobind Q Pico 0.08 mL, and 3M Polisher ST BC1 capsules. Table 1 contains product specifications for each device. Cytiva Mustang E and Sartorius Sartobind Q are membranes comprising Q functional groups. As mentioned previously, 3M Polisher ST combines a Q‐functional nonwoven with a guanidinium functional membrane within each device. 3M Polisher ST single‐use filter products are intended for use only in pharmaceutical processing applications.

### 2.2 Preparation of Endotoxin Spiked Buffers

For this study, a bracketing experimental design (MODDE by Sartoius Stedim Biotech GmbH; D‐optimal design) was chosen to examine the pH and conductivity ranges to broadly cover scenarios needed for an UFDF operation. Two buffer types were prepared: (1) a UFDF start buffer matrix and (2) UFDF diafiltration buffer matrix, including several types of substances like amino acids, sugar, mono‐ and polyvalent buffer salts. Endotoxin (*E. coli* 055:B5, Charles River Laboratories, Charleston, SC, USA) was spiked to levels that covers a worst‐case scenario, where maximum endotoxin levels of individual buffer components declared in each of their respective specification sheets are summed together and represent the theoretical maximum endotoxin concentration from the raw material inputs during this unit operation. Table 2 outlines the buffer compositions and targeted endotoxin load concentration for this study. Conductivity and pH were adjusted with sodium chloride and 5 M NaOH or 5 M HCl, respectively.

**TABLE 2:** Buffer compositions.

Buffer	Raw Material	Concentration (mM)	Endotoxin target load (EU/mL)
UFDF start buffer matrix DoE pH range: 4.7–7.7; DoE conductivity range: 6–43 mS/cm	MES, monohydrate	20	1.5
	HEPES	20	
	Acetic acid, glacial	20	
	Sodium chloride (NaCl)	20–400	
	5 M HCl/5 M NaOH	*Quantum satis*; for pH adjustment	
	Water, purified (PW+)	*Quantum satis*	
UFDF diafiltration buffer matrix DoE pH range: 4.7–8.3; DoE conductivity range: 7–22 mS/cm	(L‐)Histidine	20	1.0
	(L‐)Arginine hydrochloride	75	
	Saccharose	80	
	Tris	20	
	Citric acid monohydrate	10	
	Sodium chloride (NaCl)	0–165	
	5 M HCl/5 M NaOH	*Quantum satis*; for pH adjustment	
	Water, purified (PW+)	*Quantum satis*	

### 2.3 Chromatography Studies

Chromatography studies using the membrane adsorbers were controlled and monitored with chromatography systems (Cytiva ÄKTA Avant or Cytiva ÄKTA Pure). Preconditioning flushes were applied as needed, unless otherwise stated, as specified by the manufacturer, and stated in Table 1.

#### 2.3.1 Preconditioning Flush

Examination of the required NaOH and NaCl preconditioning flush steps on endotoxin removal was carried out using these anion exchangers. As mentioned before, 3M Polisher ST may be used without a sanitization flush according to the manufacturer's instructions; however, end users may need to perform their own risk assessment. Thus, two different sets of capsules were prepared: (1) Set 1: NaOH, NaCl, and Equilibration: Cytiva Mustang E and Sartorius Sartobind Q preconditioned with NaOH and NaCl flush steps prior to equilibration with UFDF start buffer matrix; (2) Set 2: Equilibration only: All membrane adsorbers were equilibrated with UFDF start buffer matrix. For equilibration, the UFDF start buffer matrix contained 200 mM NaCl at pH 7.0 and was not spiked with endotoxins.

The protocol used for preconditioning Cytiva Mustang E was as follows: (a) sanitization: 30 membrane volume (MV) of 1 M NaOH at 1 MV/min; (b) salt wash: 10 MV of 1 M NaCl at 10 MV/min; (c) equilibration: 30 MV of UFDF start buffer matrix containing 200 mM NaCl, pH 7.0, (without endotoxin spike) at 10 MV/min. Sartorius Sartobind Q was preconditioned in a similar manner: (a) sanitization: 30 MV of 1 M NaOH at 1 MV/min; (b) salt wash: 50 MV of 1 M NaCl at 10 MV/min; (c) equilibration: 50 MV of UFDF start buffer matrix containing 200 mM NaCl, pH 7.0, (without endotoxin spike) at 10 MV/min. Equilibration only capsules of 3M Polisher ST, Sartorius Sartobind Q, and Cytiva Mustang E were conditioned by 54 L/m^2^ at 600 LMH, 50 MV at 10 MV/min, and 30 MV at 10 MV/min, respectively, with UFDF start buffer matrix without endotoxin spike.

Following capsule preconditioning, each device was challenged with the UFDF start buffer matrix containing 200 mM NaCl, at pH 7.0, spiked up to 1.5 EU/mL of *E. coli* 055:B5 endotoxin. Cytiva Mustang E, Sartorius Sartobind Q, and 3M Polisher ST were loaded with 250 mL at 10 MV/min, 150 mL at 10 MV/min, and 200 mL at 600 LMH, respectively. For NaOH and NaCl conditioned Cytiva Mustang E and Sartorius Sartobind Q, endotoxin was quantified in each filtrate pool collected. Four filtrate fractions were collected for devices conditioned with only equilibration buffer. Filtrate fraction volumes of 50, 37.5, and 62.5 mL were collected from 3M Polisher ST, Sartorius Sartobind Q, and Cytiva Mustang E, respectively. Individual fractions and a combined pool were analyzed for endotoxin concentration.

#### 2.3.2 Bracketing Study for Endotoxin Removal From UFDF Start and Diafiltration Buffers

Capsules were loaded up to approximately 2000 mL buffer per mL of MV with solutions from Table 2. The run sequence for this designed study examining pH and conductivity was randomized. For 3M Polisher ST, the G functional membrane MV (0.14 mL) was considered. This equated to a load volume of 160, 240, and 280 mL applied to Sartorius Sartobind Q, Cytiva Mustang E, and 3 M Polisher ST, respectively. Flow rates for 3M Polisher ST, Cytiva Mustang E, and Sartorius Sartobind Q were 1.0, 1.2, and 0.8 mL/min, respectively. NaOH or NaCl flushes were not applied to the capsule flushes for these studies.

#### 2.3.3 Filtration Time Optimization

To push the boundaries of the buffer processing conditions, a maximum flux with a safety factor was employed for this time optimization study. The flux used was determined prior to this study by examining the delta pressure at a given flow rate with 3M Polisher ST BC1 capsules. The manufacturer specification for these capsules has a maximum delta membrane pressure of ≤2.4 bar. A capsule pressure study was performed on an AKTA system, showing an upper flow rate limit of 6 mL/min at 2.1 bar, allowing a pressure safety margin of 12.5%. The flux at this flow rate was 3,600 LMH for a 1 cm^2^ module. Individual fractions were taken every 1,000 L/m^2^, up to a throughput of 10,000 L/m^2^, and each fraction was analyzed for endotoxin concentration.

### 2.4 Endotoxin Quantification

Endotoxin quantification in samples was performed using kinetic turbidimetric testing, following standard methods Ph. Eur. 2.6.14 and USP <85>. In summary, the Limulus Amebocyte Lysate (LAL) test is an in vitro method for the qualitative and quantitative analysis of endotoxins, released by gram‐negative bacteria. Addition of endotoxins to LAL induces turbidity, precipitation, or gelation. The method is based on time determination, to reach a predefined absorption. Analytical variance is accepted in a range of 50%–200%, referred to a standard in accordance with the pharmacopoeia guidelines.

## 3 Results and Discussion

### 3.1 Preconditioning AEX Membranes for Endotoxin Removal

Anion exchange chromatography technologies were examined for the impact of NaOH and NaCl preconditioning flushes on endotoxin removal, based upon manufacturer instructions. The spiked buffer load was considered with respect to available module sizes. For an applicable transfer in production, we assumed a 1000 L buffer per module with a targeted guide of approximately 3 h. Since each product size specification was different (i.e., surface area vs. volume), the load was not identical for all devices but was considered applicable and comparable based on processing times.

As described in the method section, individual fractions and pooled samples were collected from processing endotoxin spiked UFDF start buffer matrix with Q1, Q2, and G1. Table 3 indicates that G1 and Q2 were able to remove >1.96 log of endotoxin to below the limit of detection from spiked buffer, while Q1 was only able to reduce the endotoxin content by 87%–88% from a UFDF start buffer matrix with a salt concentration of 200 mM NaCl at pH 7.0. Q1 was the only chromatography device showing endotoxin breakthrough at a throughput after the first 0.5 L/mL throughput (data not shown). As expected, the data showed pre‐flushing capsules with NaOH and NaCl did not appear to impact the removal of endotoxins from buffers since both Q membranes examined are known to be compatible with these solutions. Base sanitization was not performed with the G membrane device but the regulatory support file shows bovine serum albumin dynamic binding was not reduced by sanitization procedures, 1 M NaOH for up to 1 h, or autoclaving, 121°C for 30 min, compared to an untreated control. Additionally, these chromatographic solutions did not impact the buffer composition as chromatograms from each membrane adsorber maintained stable conductivity and pH values throughout the filtration process (data not shown).

**TABLE 3:** Endotoxin level after processing UFDF start buffer matrix (200 mM NaCl, pH 7.0).

AEX chromatographic solution	Capsules pre‐flushed with 1 M NaOH and 1 M NaCl	Capsule loading	Time/run (min)	Endotoxin level in pooled filtrate (EU/mL)
UFDF Start Buffer Matrix				0.92^a^
Cytiva Mustang E (Q1)	Yes	2.083 L/mL	228	0.12
	No	2.083 L/mL	228	0.11
Sartorius Sartobind Q (Q2)	Yes	1.852 L/mL	242	<0.01^b^
	No	1.852 L/mL	242	<0.01
3M Polisher ST (G1)	No	2,000 L/m^2^	205	<0.01

^a^ 0.92 EU/mL corresponds to a recovery rate of 61% referred to the theoretical endotoxin spike of 1.5 EU/mL.

^b^ Limit of detection in dependency of sample dilution.

### 3.2 Influence of Buffer Composition on Endotoxin Removal

Although the charge capacity was not measured for each device, the ionic charge capacity for all devices should be more than sufficient to remove the total amount of endotoxin exposed through each buffer matrix tested based on protein or endotoxin binding capacity studies described in product literature by the manufacturers and shown in Table 1. Devices were challenged up to a total of 3 ×10^3^ EU per mL of membrane, which falls well below the reported or calculated endotoxin binding capacity gathered from the manufacturer's application notes and product literature of greater than 1 ×10^6^ EU per mL of membrane for both Q1 and Q2. G1 should have enough capacity as well, since it has about 1.4 times more BSA dynamic binding capacity than that of Q2 based on the bed volume.

Tables 4 and 5 indicate endotoxin concentrations in filtrates from membrane adsorbers challenged with UFDF start buffer and diafiltration buffer, respectively. Endotoxin removal by Q‐based membrane adsorbers challenged with the UFDF start buffer was reduced by increasing salt concentrations, as shown in Table 4, but not necessarily by pH. Q1 performance was impacted by salt concentration, showing endotoxin removal to the limit of detection at conductivities around 6 to 8 mS/cm while showing less efficacy at higher conductivities. Even though Q1 and Q2 have similar Q‐chemistry, Q2 showed slightly higher salt tolerance up to 24 mS/cm for endotoxin removal. UFDF start buffer processed by G1 was observed to remove endotoxin up to a conductivity of 43 mS/cm across a pH range of 4.7 to 7.7 compared to the Q‐functional adsorbers. The difference between the Q functional membranes may be due to the ligand chemistry, charge density, cross‐link density, membrane characteristics, or combinations thereof, and directly influence how these materials interact with endotoxin. Computational studies investigating the interactions between AEX sorbents and phosphorylated endotoxin residues revealed that the spacer group, which is the chemical group used to covalently attach the AEX ligand to a substrate, often cooperatively interacts with the endotoxin molecule [24]. Additional computational modeling studies could further elucidate the effect of ligand type and charge density on binding endotoxin molecules by examining various multimer or polymeric ligands, including spacer chemistry, and applying environmental conditions.

**TABLE 4:** Bracketing DoE for UFDF start buffer matrices.

AEX chromatographic solution	pH	NaCl (mM)	Conductivity (mS/cm)	Endotoxin (EU/mL)
UFDF Start Buffer				0.94^a^
Cytiva Mustang E (Q1)	4.7	20	6	<0.02^b^
	7.7	20	8	<0.02
	4.7	400	43	0.39
	7.7	400	41	0.35
	6.2	210	24	0.39
Sartorius Sartobind Q (Q2)	4.7	20	6	<0.02
	7.7	20	8	<0.02
	4.7	400	43	0.27
	7.7	400	41	0.82^c^
	6.2	210	24	<0.02
3M Polisher ST (G1)	7.7	20	8	<0.02
	4.7	400	43	<0.02
	4.7	146.7	18	<0.02
	7.7	273.3	31	<0.02
	5.7	20	7	<0.02
	6.7	400	42	<0.02
	6.2	210	24	<0.02
	6.2	210	24	<0.02
	6.2	210	24	<0.02

^a^ 0.94 EU/mL corresponds to a recovery rate of 63% referred to the theoretical endotoxin spike of 1.5 EU/mL.

^b^ Limit of detection in dependency of sample dilution.

^c^ Average of duplicate measurement to confirm higher endotoxin level.

**TABLE 5:** Bracketing DoE for UFDF diafiltration buffer matrices.

AEX chromatographic solution	pH	NaCl (mM)	Conductivity (mS/cm)	Endotoxin (EU/mL)
UFDF Diafiltration Buffer				1.14^a^
Cytiva Mustang E (Q1)	8.3	165	21.6	0.12
	4.7	55	12.9	0.21
	4.7	110	17.4	0.18
	8.3	55	12.3	0.02
	5.9	0	7.16	0.07
	7.1	0	7.00	0.04
	5.9	165	21.3	0.12
	6.5	82.5	14.0	0.27
Sartorius Sartobind Q (Q2)	4.7	0	7.52	<0.01^b^
	8.3	0	8.79	<0.01
	4.7	165	21.8	0.25
	8.3	165	21.6	<0.01
	6.5	82.5	14.0	<0.02
	6.5	82.5	14.0	<0.02
	6.5	82.5	14.0	<0.02
3M Polisher ST (G1)	4.7	0	7.52	<0.01
	8.3	0	8.79	<0.01
	4.7	165	21.8	<0.01
	8.3	165	21.6	<0.01
	6.5	82.5	14.0	<0.02

^a^ 1.14 EU/mL corresponds to a recovery rate of 114% referred to the theoretical endotoxin spike of 1.0 EU/mL.

^b^ Limit of detection in dependency of sample dilution.

**FIGURE 1:**
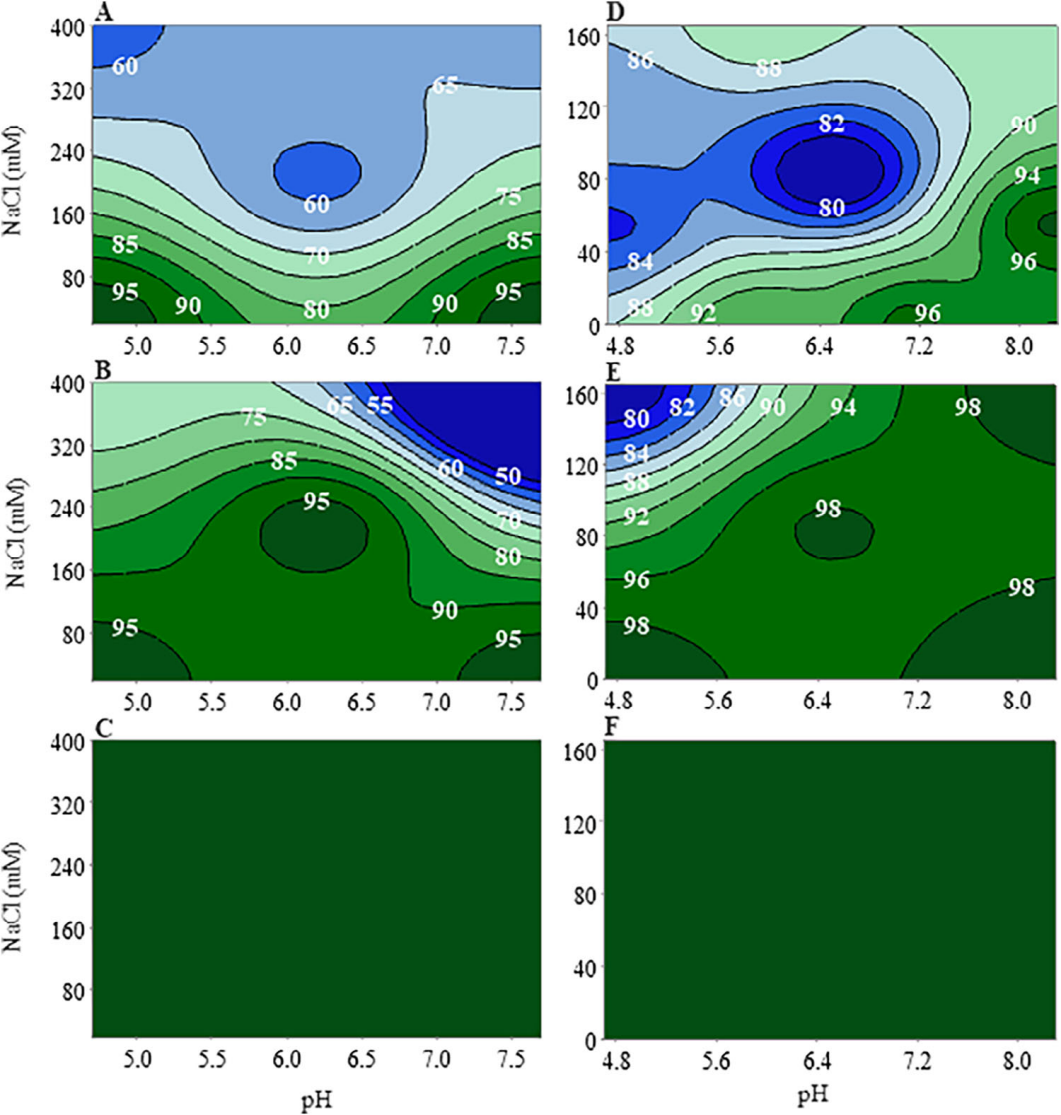
Percent endotoxin reduction by anion exchangers as a function of salt concentration vs. pH. Response contour plots of UFDF starting buffer matrix processed by (A) Cytiva Mustang E, (B) Sartorius Sartobind Q, and (C) 3M Polisher ST showing >95% endotoxin reduction in dark green and decreasing to <50% in dark blue. Endotoxin reduction in terms of percentage for UFDF diafiltration buffer matrix processed by (D) Cytiva Mustang E, (E) Sartorius Sartobind Q, and (F) 3 M Polisher ST indicate <80% endotoxin reduction in dark blue and increasing to greater than 98% in dark green. White numbers in plots indicate the percent endotoxin reduction associated at each contour line.

For diafiltration buffer matrices comprising a pH of 4.7–8.3 and conductivity of 7.0–21.6 mS/cm, Q1 showed endotoxin concentrations above the limit of detection (<0.01 EU/mL), ranging between 0.02 and 0.21 EU/mL. Q2 and G1 processed solutions showed endotoxin levels below the limit of detection except for Q2 at one buffer condition (pH 4.6, 21.8 mS/cm). Figure 1 visually represents the performance of these devices by showing the endotoxin reduction response to buffer pH and conductivity. Figure 1 was based on information in Tables 4 and 5 and generated by Minitab (V20.4).

The variation in performance of Q membrane adsorbers between the UFDF start and diafiltration buffers appears to be due to their composition. Transitioning to a more complex buffer matrix, including drug‐stabilizing compounds such as histidine and arginine, associated with the UFDF diafiltration buffer, could mask interactions with the adsorbers. Additionally, buffers containing multivalent salts, such as phosphate or citrate, are generally known to reduce the binding capacity of AEX membranes adsorbers. Histidine immobilized on filtration media and histidine tags on proteins have been known to adsorb endotoxin [15, 25, 26]. Histidine by itself can carry a positive charge at a pH below its pKa of approximately 6. Arginine, a G containing amino acid, has been used to remove protein‐associated endotoxin, theorized by neutralizing the negatively charged lipid A moiety [27]. Investigation of amino acid ligands immobilized onto agarose beads through a diaminohexane spacer demonstrated that arginine had the highest endotoxin adsorption capacity of the 10 amino acids tested [24]. Other G ligands immobilized onto membranes have been shown to be effective at removing negatively‐charged impurities such as viruses, host cell proteins, and DNA [28]. Thus, the presence of arginine or histidine could inhibit the ability of endotoxin binding to the membrane by interacting with the endotoxin molecule. These additional components may have led to Q1 and Q2 being more susceptible to endotoxin breakthrough by masking the interaction between the functional ligands of the membrane adsorbers and endotoxin. This might explain why Q2 shows endotoxin breakthrough processing the diafiltration buffer matrix containing histidine at pH 4.7 and not at pH 8.3 in buffers containing 165 mM NaCl (∼21 mS/cm), as histidine may have a stronger interaction with endotoxin than the Q‐endotoxin interaction at that buffer condition. Additional experimentation by challenging membranes with individual buffer components, such as histidine, in the presence of endotoxin, may elucidate this observation.

Under all conditions tested, the G membrane adsorber endotoxin removal performance was not diminished by the presence of these components or under high salt conditions. The Q‐functional nonwoven apart of G1 can bind endotoxin under lower salt conditions, but the guanidinium membrane would become the predominant binding adsorber for endotoxin in higher salt conditions. This performance can partially be explained by the guanidinium ligands having a high net positive charge to outcompete buffer salt components and form stable salt bridges with oxoanions, such as carboxylate residues in proteins or phosphate‐containing molecules like endotoxin [23, 29, 30]. The salt bridges, comprised of electrostatic interaction and two‐hydrogen bonding interactions, lead to exceptional binding capacities in high salt conditions. These attributes, along with the previous mentioned research on guanidinium‐functionalized membranes, indicate these materials are extremely sticky toward negatively charged molecules [28].

One example of a primary amine adsorber is a membrane functionalized with polyallylamine. Salt‐tolerant polyallylamine membranes have demonstrated high endotoxin removal from buffer up to 250 mM NaCl [19, 31]. However, both PA and Q chemistries lack the ability to form bidentate hydrogen bonds outside of their electrostatic forces [23]. However, polyallylamine membranes have been shown to be less affected by binding protein and viruses in the presence of higher monovalent salt concentrations but appear significantly more impacted by the presence of multivalent salts compared to a Q membrane adsorber [32]. Understanding the impact of solution compositions on removal performance is critical in determining which membrane adsorber will fit the user's needs.

### 3.3 Application: Time Optimization of UFDF Start Buffer Matrix Processing

Optimization of buffer processing for filtration time was considered for this study to ensure endotoxin reduction under challenging conditions. Since the G membrane adsorber showed the highest salt tolerance, G1 was processed under challenging conditions with endotoxin containing UFDF start buffer matrix (pH 6.2) containing 400 mM NaCl (conductivity 41.8 mS/cm) up to approximately 10,000 L/m^2^ at a flux of 3,600 LMH. An endotoxin spike recovery was determined to be 71% from the start buffer matrix. Results showed the limit of quantification for endotoxin (<0.1 EU/mL) in each fraction up to 10,000 L/m^2^. Overall, endotoxin was reduced to the limit of detection or by greater than 93.9% (>1 log).

## 4 Concluding Remarks

AEX membrane adsorbers were an effective technique to remove residual levels of endotoxin from buffer solutions. A bracketing design study to examine effective removal was critical to understand the effect of buffer properties and process conditions to successfully deplete of endotoxin using each AEX membrane adsorber. Cytiva Mustang E demonstrated effective endotoxin removal from a basic UFDF buffer composition with low conductivity. Even though Sartorius Sartobind Q and Cytiva Mustang E use similar Q‐based chemistry immobilized on a membrane, Sartorius Sartobind Q demonstrated a higher salt tolerance and endotoxin binding in the presence of different buffer compositions than Cytiva Mustang E. 3M Polisher ST demonstrated endotoxin reduction to concentrations below the detection limit across the broad pH and conductivity ranges from both buffer compositions examined in this study. Overall, these membrane technologies can provide a robust approach to endotoxin removal during drug manufacturing, but a thoughtful approach must be considered to identify their limitations and effective processing conditions.

## Conflicts of Interest

All authors were employees of Solventum (formerly 3M Health Care) and Rentschler Biopharma SE at the time the work was performed. Sophie Muczenski and Andrew Vail are employees of Solventum, the corporation that developed and produces 3M(TM) Polisher ST and 3M(TM) Emphaze AEX Hybrid Purifier. Sven Heimann and Alexander Faude are employees of Rentschler Biopharma SE, and they designed and conducted this study independently.

## Data Availability

The data that support the findings of this study are available from the corresponding author upon reasonable request.
